# Interference-Aware Subcarrier Allocation for Massive Machine-Type Communication in 5G-Enabled Internet of Things

**DOI:** 10.3390/s19204530

**Published:** 2019-10-18

**Authors:** Wenjun Hou, Song Li, Yanjing Sun, Jiasi Zhou, Xiao Yun, Nannan Lu

**Affiliations:** 1 School of Information and Control Engineering, China University of Mining and Technology, Xuzhou 221000, China; hauomenguanu@cumt.edu.cn (W.H.); lisong@cumt.edu.cn (S.L.); jiasi_zhou@cumt.edu.cn (J.Z.); yx.tong@163.com (X.Y.); lunannan@cumt.edu.cn (N.L.); 2 school of Communication and Information Engineering, Xi’an University of Science and Technology, Xi’an 710054, China

**Keywords:** 5G, internet of things, mMTC, eMBB

## Abstract

Massive machine-type communication (mMTC) is investigated as one of three typical scenes of the 5th-generation (5G) network. In this paper, we propose a 5G-enabled internet of things (IoT) in which some enhanced mobile broadband devices transmit video stream to a centralized controller and some mMTC devices exchange short packet data with adjacent devices via D2D communication to promote inter-device cooperation. Since massive MTC devices have data transmission requirements in 5G-enabled IoT with limited spectrum resources, the subcarrier allocation problem is investigated to maximize the connectivity of mMTC devices subject to the quality of service (QoS) requirement of enhanced Mobile Broadband (eMBB) devices and mMTC devices. To solve the formulated mixed-integer non-linear programming (MINLP) problem, which is NP-hard, an interference-aware subcarrier allocation algorithm for mMTC communication (IASA) is developed to maximize the number of active mMTC devices. Finally, the performance of the proposed algorithm is evaluated by simulation. Numerical results demonstrate that the proposed algorithm outperforms the three traditional benchmark methods, which significantly improves the utilization of the uplink spectrum. This indicates that the proposed IASA algorithm provides a better solution for IoT application.

## 1. Introduction

In the future industrial internet of things (IIoT), a large number of devices including monitoring sensors and execution control units will be deployed to support factory automation and industry control system [1]. Massive periodic/non-periodic data will be transferred to a centralized control unit or adjacent devices via an industry wireless network, including video monitoring information, sensing data, operation instructions. However, due to the limited capacity and throughput of the current cellular system, it is insufficient in supporting future IoT applications with a tremendous number of devices and heterogeneous information traffic [2].

Massive machine-type communication (mMTC), as one of three typical application scenarios in the 5th-generation (5G) network, is investigated to support communication among a massive number of devices, which provides a feasible solution for future industrial IoT (IIoT) [3]. Due to limited spectrum resources in the cellular system, massive devices access the wireless network in a spectrum-sharing manner in which multiple devices are allocated in the same spectrum at the same time. Thus, the co-channel interference among devices restricts the number of devices connected to the cellular system. Effective interference management plays a vital role in mMTC to support the simultaneous access of more devices. The features and challenges of mMTC in IoT are as follows. First, devices in IoT need to exchange information with their neighbor devices frequently. In other words, the communication is performed between adjacent devices [4]. Second, the coding blocklengths for IoT are usually short, to reduce the transmission delay. The transmission rate cannot be estimated by the conventional Shannon’s capacity, which assumes an infinite blocklength [5]. Third, a massive number of devices in IoT need to be supported. Thus, efficient resource allocation in mMTC needs to be investigated to address these challenges.

A wide range of works have contributed to the resource allocation problem in mMTC. In [6], the authors establish an interference model and a formulate resource allocation problem between users and machine-type communication (MTC) gateways in mMTC burst scenarios. In [7], the authors investigated the access management issues for MTC devices with heterogeneous quality of service (QoS) in the same cellular network. This work does not consider bandwidth utilization because transmission opportunities are reserved for a group of MTC devices at the same time. The authors in [8] propose two relay schemes and transmission protocols to specifically stimulate system capacity. In a multi-cell MTC system, Kwon et al. [9] establishes the interference model and analyzes the signal-to-interference-plus-noise-ratio (SINR) distributions and drives efficient resource allocation schemes. In [10], the authors propose a dynamic resource allocation algorithm based on the estimation of the number of MTC devices to handle massive and dynamic MTC devices while satisfying the random access delay requirement of MTC devices. To achieve effective resource utilization, a resource allocation metric based on statistical priority is proposed in  [11]. In this way, effective resource utilization is achieved by letting MTC devices send a reduced set of their data. In [12], the authors consider a connectivity maximization problem for narrowband IoT with non-orthogonal multiple-access (NOMA). However, articles [6,7,8,9,10,11,12] assume that all devices communicate with the base station or centralized controller directly and do not consider the communications between adjacent devices.

D2D technology, as another promising technology in 5G, can establish communication between adjacent nodes, which can improve the spectrum efficiency and offload the load of Base Station (BS). D2D communication is introduced to mMTC system to stimulate spectrum efficiency and support more mMTC devices accessed with limited spectrum resources. However, designing better resource allocation algorithms to manage the inter-user interference between D2D users and cellulars is the key challenge for improving system performance. Resource allocation and interference problems of D2D communication have been investigated in many works [13,14,15]. The authors in [16] propose a cell sectorization scheme to alleviate the interference between cellular users and D2D users. In [17], the authors investigate interference coordination for downlink full-dimension multiple-output systems with underlying D2D communications.

Adopting D2D technology, the number of supported devices can be improved in mMTC scenarios [18]. By allowing unauthorized devices to reuse the frequency bands of authorized cellular users, bandwidth utilization can be improved [19,20,21]. The literature  [22] proposes a heuristic subcarrier allocation method to set the user’s signal-to-noise ratio (SNR) threshold to meet the QoS of the system. The authors in [23] propose a mobile traffic offloading scheme that combines small base stations with D2D offloading. The goal is to accommodate a large number of MTC connections by maximizing the throughput of the network system. The impact of radio frequency energy harvesting on the spectral efficiency of the D2D-assisted MTC system is analyzed first in [24]. In [25], the authors propose two solutions to manage the communication between D2D devices and the BS to lighten the overhead of MTC devices on the 5G network. However, resource allocation [19,20,21,22,23,24,25] mainly focuses on throughput maximization or interference minimization. In an IIoT enabled by D2D communications, massive devices demand access to the network via D2D mode. Thus, the connectivity maximization problem becomes a challenging issue to tackle. To support a system in which the number of users is higher than the number of subcarriers, a range of fair subcarrier allocation algorithms is proposed that always improves the reliability [26]. However, the author only considers the scenario of mobile users in the downlink and does not consider the influence of interference.

In this paper, we investigate a D2D-enabled internet of things in which some devices (enhanced Mobile Broadband (eMBB) devices) connect to the centralized controller while other devices (mMTC devices) communicate with their adjacent devices via D2D communication to promote inter-device cooperation in industry automation. Specifically, mMTC devices reuse the spectrum resource with eMBB devices. We establish the connectivity maximization problem of mMTC devices while guaranteeing the QoS of eMBB devices and mMTC devices. Furthermore, we propose an interference-aware subcarrier allocation algorithm to tackle the problem. The main contributions of this paper are as follows:

- We establish a problem of maximizing the number of accessed mMTC pairs subject to the constraints of QoS in a system with both eMBB and mMTC devices, which is proven to be a mixed-integer non-linear programming (MINLP) problem.
- We propose an interference-aware subcarrier allocation algorithm for mMTC (IASA) considering the interference range of each mMTC device.
- In order to evaluate the proposed algorithm, a simulation is conducted. The results demonstrate that the proposed algorithm outperforms two benchmark algorithms significantly in terms of the number of mMTC pairs accessed under the same constraints.

The remainder of the paper is organized as follows. The system model and assumptions is elaborated in the “System Model” section. The optimization problem and constraints are introduced in the “Problem Formulation” section. The proposed subcarrier allocation algorithm is presented in the “Interference-Aware Subcarrier Allocation for mMTC Communication Algorithm” section. Comprehensive simulation results are provided in the “Simulation result analysis” section. Finally, we conclude the paper in the ”Conclusion” section.

## 2. System Model

**Figure 1:**
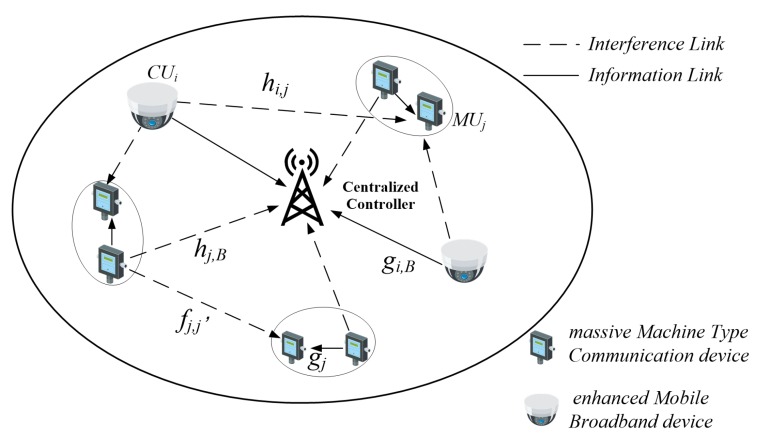
System model.

In this paper, we investigate an industrial wireless network in which some devices, referred to as eMBB devices (such as monitoring cameras), transmit video information to a centralized controller, while devices referred to as mMTC devices (such as sensors and actuators), transmit short blocklength packets to their adjacent devices to promote inter-device cooperation and industrial automation. The system model is illustrated in Figure 1, where *N* eMBB devices and *M* mMTC devices are randomly distributed, represented by sets $N=\left\{CU_{1,\ldots ,}CU_{i,\ldots ,}CU_{N}\right\}$ and $M=\left\{MU_{1,\ldots ,}MU_{j,\ldots ,}MU_{M}\right\}$, respectively. The mMTC devices transmit information from the transmitters to receivers by the D2D communication mode, and the mMTC pairs reuse the eMBB devices’ uplink resources in order to improve the spectrum efficiency. The mMTC pairs and eMBB devices are represented by $MU_{j}$ and $CU_{i}$, respectively. Each mMTC pair is composed of one mMTC transmitter and one mMTC receiver represented by $MU_{j}^{t}$ and $MU_{j}^{r}$, respectively. All available spectrum resources are divided into sub-carriers with the same bandwidth. Each eMBB device occupies mutually orthogonal sub-carriers. Therefore, there is no co-channel interference between the eMBB devices. We assume that the eMBB device $CU_{i}$ occupies the subcarrier *i*. Considering the impact of devices on each other, each mMTC pair is allowed to access no more than one subcarrier, and each subcarrier can be accessed by multiple mMTC devices. All of the channels in the system are assumed to be quasi-static Rayleigh fading channels. The channel gain remains constant for each symbol transmission period but varies independently between different symbol periods. The parameters in the article are shown in Table 1.

We define a binary subcarrier allocation matrix $A\in {\left\{0,1\right\}}^{M\times N}$, where $\alpha_{ij}=1$ indicates that $MU_{j}$ occupies subcarrier *i*, otherwise $\alpha_{ij}=0$, i∈N, j∈M. Thus, the received signal of the base station on subcarrier *i* is

(1) $$y_{i}=\sqrt{P_{i}^{c}}g_{i,B}x_{i}+\sum_{j\in M}\alpha_{ij}\sqrt{P_{j}^{d}}h_{j,B}x_{j}+n_{0},$$

where $x_{i}$ and $x_{j}$ are the transmitted signals of the eMBB devices $CU_{i}$ and the mMTC transmitter $MU_{j}^{t}$, respectively. $n_{0}$ represents normalized additive white Gaussian noise, $n_{0}∼CN\left(0,\sigma_{0}^{2}\right)$. When the centralized controller receives the uplink signals of the eMBB device $CU_{i}$, the SINR at centralized controller can be calculated as

(2) $$\gamma_{cu}^{i}=\frac{{\left|g_{i,B}\right|}^{2}P_{i}^{c}}{I+\sigma_{0}^{2}B},$$

where *B* is the subcarrier bandwidth and *I* represents the interference caused by the mMTC pairs which access the subcarrier *i*:

(3) $$I=\sum_{j=1}^{M}\alpha_{ij}P_{j}^{d}{\left|h_{j,B}\right|}^{2}.$$

Since the eMBB devices transmit long packet data, the achievable transmission rate of the eMBB device $CU_{i}$ can be obtained by Shannon’s theorem:

(4) $$R_{cu}^{i}=B\log\left(1+\gamma_{cu}^{i}\right).$$

The signal received by the *j*th mMTC pair $MU_{j}$ is

(5) zj=Pjdgjxj+∑i∈NαijPichi,jxi+∑j′=1j′≠jM∑i∈Nαij′Pj′dfj′,jxj′+n0,

where the first term is the signal receiver $MU_{j}^{r}$ received from mMTC transmitter $MU_{j}^{t}$. The second term is the interference from the eMBB device $CU_{i}$. The third term is the interference signal from the transmitter of the mMTC pair $MU_{j^{\prime }}$ that occupies the same subcarrier with mMTC pair $MU_{j}$. According to (5), the SINR of the mMTC receiver $MU_{j}^{r}$ can be derived as

(6) γj=Pjdgj2∑i∈NαijPichi,j2+∑j=1j′≠jM∑i∈Nαij′Pj′dfj′,j2+σ02.

Since mMTC communication is mostly aimed at periodic monitoring data in IoT applications such as smart cities, the length of data packets transmitted is usually very short. According to information theory, the rate of short packets cannot achieve the Shannon limit. Therefore, the transmission rate of the mMTC devices is represented by the short packet rate [27], as shown in (7):

(7) $$R_{j}=\log_{2}\left(1+\gamma_{j}\right)-\sqrt{\frac{1}{m}}\log_{2}\left(e\right)Q^{-1}\left(\varepsilon \right)\sqrt{1-\frac{1}{{\left(1+\gamma_{j}\right)}^{2}}}$$

where *m* is the block length, ε is the transmission error probability, and $Q^{-1}\left(x\right)$ is the inverse of the Gaussian *Q* function.

## 3. Problem Formulation

The optimization goal of this paper is to maximize the total number of mMTC devices accessed under the QoS of each eMBB device. All of the mMTC devices access the network adhere to the following criteria:

(i) Each mMTC device is allowed to access no more than one subcarrier.
(ii) To ensure the transmission quality of the eMBB devices, the interference each subcarrier can suffer should be below a threshold.
(iii) Both eMBB devices and mMTC devices should satisfy their own transmission rate requirements.

When the *j*th mMTC pair is allowed to access the subcarrier *i*, the SINR of the receiver of $MU_{j}$ is

(8) γj=Pjdgj2Pichi,j2+∑j′=1j≠jMαij′Pj′dfj′,j2+σ02.

Taking $\gamma_{j}$ in (8) into (7), we can get the transmission rate $R_{j}$ after mMTC pair $MU_{j}$ is allowed to access the subcarrier *i*. In order to ensure the QoS of the mMTC pair $MU_{j}$, the achievable rate $R_{j}$ of the mMTC pair $MU_{j}$ should not be less than the minimum required rate $R_{j_{-}\min}$, i.e.,

(9) $$R_{j}\geq R_{j_{-}\min}.$$

When the mMTC pair $MU_{j}$ is allowed to access the subcarrier *i*, the minimum transmission rate of the eMBB device $CU_{i}$ is $R_{cu_{-}\min}^{i}$. Under the QoS constraint of the eMBB devices and the mMTC pairs, we maximize the number of mMTC pairs accessed in the network. Mathematically, the optimization problem is formulated as

(10a) $$\begin{array}{cc} \; & \max_{\alpha_{ij}}\sum_{i=1}^{N}\sum_{j=1}^{M}\alpha_{ij}\;, \end{array}$$

(10b) $$\begin{array}{cc} s.t\; & R_{cu}^{i}\geq R_{cu_{-}\min}^{i}\;, \end{array}$$

(10c) $$\begin{array}{cc} & R_{j}\geq R_{j_{-\min}}\;, \end{array}$$

(10d) $$\begin{array}{cc} & \alpha_{ij}\in \left\{0,1\right\},\;\forall i\in N\;j\in M\;, \end{array}$$

(10e) $$\begin{array}{cc} & \sum_{i=1}^{N}\alpha_{ij}\leq 1. \end{array}$$

The constraints described in (10b) and (10c) indicate that the respective minimum transmission rate requirements of the eMBB devices and the mMTC pairs should be satisfied. Equation (10e) reveals that each mMTC pair is allowed to occupy at most one subcarrier. The optimization problem is a binary optimization problem, and the traversal complexity of the problem is $2^{MN}$. In the following section, we propose a lower complexity algorithm named interference-aware subcarrier allocation for mMTC communication.

## 4. Interference-Aware Subcarrier Allocation for mMTC Communication Algorithm

According to the constraint condition (10b), the accumulated interference allowed by the eMBB device $CU_{i}$ can be derived from the minimum transmission rate $R_{cu_{-}\min}^{i}$

(11) $$I_{cu_{-}\max}^{i}=\frac{P_{i}^{c}{\left|g_{i,B}\right|}^{2}}{2^{R_{cu_{-}\min}^{i}}-1}-\sigma_{0}^{2}B.$$

When the *j*th mMTC pair $MU_{j}$ occupies the subcarrier *i*, the interference caused by $MU_{j}$ to the base station is

(12) $$I_{j,B}=P_{j}^{d}{\left|h_{j,B}\right|}^{2}.$$

To ensure the rate requirement of the eMBB device, the interference caused by the mMTC pair $MU_{j}$ should not exceed the maximum interference allowed by the subcarrier *i*, i.e.,

(13) $$I_{j,B}\leq I_{cu_{-}\max}^{i}.$$

Therefore, a set of mMTC pairs allowed to occupy subcarrier *i* can be selected according to Equation (13). For all mMTC pairs that can be accessed, we first define the normalized interference caused by each pair as $I_{j,i}=I_{j,B}/I_{cu_{-}\max}^{i}$. Define the interference matrix as

$$\Omega =\left[\begin{array}{cccc} I_{1}^{1} & I_{2}^{1} & \cdots & I_{M}^{1}\\ I_{1}^{2} & I_{2}^{2} & \cdots & I_{M}^{2}\\ \vdots & \vdots & \cdots & \vdots \\ I_{1}^{N} & I_{2}^{N} & \cdots & I_{M}^{N} \end{array}\right].$$

When the mMTC pair $MU_{j^{\prime }}$, $j^{\prime }\neq j$, attempts to access the subcarrier *i*, the following two conditions should be satisfied.

(i) The interference caused by $MU_{j^{\prime }}$ and the total interference of mMTC pairs cannot exceed the maximum interference allowed by subcarrier *i*, i.e., $I_{cu_{-}\max}^{i}$.
(ii) All of the accessed mMTC pairs should satisfy their own QoS.

When both conditions are satisfied, the mMTC pair is allowed to access the subcarrier. In order to facilitate the calculation, we convert the rate constraint of the eMBB device in (11) into an interference limit:

(14) $$I_{cu}^{i}\leq I_{cu_{-}\max}^{i},$$

where

(15) $$I_{cu}^{i}=\sum_{j\in M}I_{j,B}\;\forall i\in N.$$

Substituting (14) for (11), then the optimization problem is converted into

(16a) $$\begin{array}{cc} & \max_{\alpha_{ij}}\sum_{i=1}^{N}\sum_{j=1}^{M}\alpha_{ij}\;, \end{array}$$

(16b) $$\begin{array}{cc} s.t\; & I_{cu}^{i}\leq I_{cu_{-}\max}^{i}\;, \end{array}$$

(16c) $$\begin{array}{cc} & R_{j}\geq R_{j_{-}\min}\;, \end{array}$$

(16d) $$\begin{array}{cc} & \alpha_{ij}\in \left\{0,1\right\},\;\forall i\in N\;j\in M\;, \end{array}$$

(16e) $$\begin{array}{cc} & \sum_{i=1}^{N}\alpha_{ij}\leq 1\;\forall j\in M. \end{array}$$

The optimization variables of this problem are all binary variables, and the optimization problem (16a) is an NP-hard problem that cannot be solved by the convex optimization method. The traditional way to solve an NP-hard problem is to carry out an exhaustive search, which involves unaccepted computational complexity. Thus, it is difficult to obtain the optimal result by direct solution. This paper proposes a heuristic algorithm with lower complexity to tackle the problem, referred to as interference-aware subcarrier allocation for mMTC.

Firstly, the mMTC pair with minimal interference to the base station is selected in the two-dimensional matrix Ω, and the access conditions (16b) and (16c) are updated according to the QoS of the eMBB devices and other mMTC pairs. Once an mMTC pair occupies a subcarrier, the mMTC pair is prohibited from accessing other subcarriers. When the sum of the interference ratio accumulated on subcarrier *i* is not less than 1, other mMTC pairs will be no longer allowed to access the subcarrier *i*.

For the constraint condition (16c), we can estimate whether the QoS requirements are still satisfied after each mMTC pair occupies the subcarrier according to (7) and (9). Due to the interference between mMTC pairs accessing the same channel, the interference range of the mMTC pair is defined in this paper to suppress interference between mMTC pairs. The interference range of an mMTC pair $MU_{j}$ is defined as the range in which the mMTC pairs suffer from the interference of $MU_{j}$. Thus, the mMTC pairs in the interference range of $MU_{j}$ cannot access the same subcarrier with $MU_{j}$ to avoid interference. In other words, when mMTC pair $MU_{j}$ accesses subcarrier *i*, the other pairs within the interference range of $MU_{j}$ cannot access subcarrier *i* to reduce the interference between the mMTC pairs and to ensure the QoS of each mMTC pair.

The IASA algorithm is summarized in Algorithm 1. Lines 1–5 of the algorithm calculate the maximum interference that all subcarriers can support (line 3) and the proportion of the interference from each mMTC pair (line 5). Then a two-dimensional matrix is formed. Lines 6–12 of the algorithm sort the data in the two-dimensional matrix in ascending order one-dimensionally. Firstly, we find the mMTC pair and subcarrier corresponding to the minimum interference ratio. Then we estimate whether the accumulated interference caused by the mMTC pair exceeds the maximum interference allowed by the subcarrier so as to determine whether the mMTC pair can access the sub-carrier (line 7). After the mMTC pair $MU_{j}$ accesses subcarrier *i*, the mMTC pair within the interference range of $MU_{j}$ is prohibited from accessing subcarrier *i* (line 9).

**Algorithm 1** Interference-Aware Spectrum Allocation for mMTC Communication Algorithm.
1:Initialize: *B*, $P_{i}^{c}$, $P_{j}^{d}$, $R_{cu_{-}\min}^{i}$, $R_{j}$, $\alpha_{ij}=0$∀i∈N, j∈M.2:Calculate $I_{cu_{-}\max}^{i}$, i∈N in accordance with (11).3:Calculate the interference $I_{j,B}$ caused by all mMTC pairs accessing subcarriers according to (10c).4:Calculate the proportion of all mMTC pairs to the maximum interference that each subcarrier can withstand $I_{cu_{-}\max}^{i}$ and obtain the interference matrix Ω.5:Select the smallest element $\left(i^{*},j^{*}\right)$ in matrix Ω. For $CU_{i}^{*}$, $MU_{j}^{*}$, judge whether (9) and (14) is established.6:If $I_{cu}^{i}\leq I_{cu_{-}\max}^{i}$, the mMTC pair is allowed to access subcarriers, $\alpha_{ij}=1$. If $I_{cu}^{i}>I_{cu_{-}\max}^{i}$, the mMTC pair is not allowed to access the subcarrier, $\alpha_{ij}=0$.7:$\alpha_{i^{\prime }j}=0,\forall i^{\prime }\in N,i^{\prime }\neq i$.8:Filter out the mMTC pair set ***J*** within the pair $MU_{j}$ interference range. Assign all the $\alpha_{ij}$   j∈J corresponding to subcarrier *i* to 0.9:Assign the ratio Ω(:,j) of the mMTC pair to the corresponding subcarrier in this cycle to s(s≥1).10:Repeat 6–9 until all of the mMTC pairs have been assigned.11:Output matrix A.


The IASA algorithm is a centralized algorithm that can be implemented by a centralized controller. First, the centralized controller collects the information from mMTC devices who want to transmit packets with their neighborhood devices, including the channel state information and the required transmission rate. Then the centralized controller obtains the spectrum allocation results according to the IASA algorithm and broadcasts the allocation results to each mMTC pair. Then each mMTC transmitter completes the packets transmission on its allocated subcarrier.

## 5. Simulation Result Analysis

In this section, we present numerical results to verify the performance of the proposed IASA algorithm. We compare the number of mMTC pairs accessing the network successfully according to the proposed subcarrier allocation algorithm, the random access algorithm, and the sequential access algorithm. The two benchmark algorithms are described as follows.

Random access algorithm (RAA): Firstly, an interference ratio is randomly selected in the two-dimensional interference matrix. Then, we find out the corresponding mMTC devices and the access to subcarrier *i*. According to (9) and (14), it can be judged whether the QoS of the eMBB devices and the mMTC devices is satisfied, that is, whether the mMTC devices can access the subcarrier *i*. In the end, we repeat the above selection and access process until all subcarriers achieve the limit of interference they can sustain.

Sequential access algorithm (SAA): All of the mMTC devices sequentially judge whether the QoS of the eMBB devices and the mMTC devices are satisfied. When the sum of the interference ratio of subcarrier *i* is more than 1, other mMTC devices are prohibited from accessing the subcarrier *i*.

Greedy algorithm (GA): In the greedy algorithm, each subcarrier gives priority to its own access number. Specifically, the mMTC pair with minimum interference to a certain subcarrier is firstly accessed, when the QoS of the eMBB device and mMTC pair can be satisfied. When the cumulative interference of subcarrier *i* exceeds 1, other mMTC pairs are forbidden from accessing the subcarrier.

It is assumed that the eMBB and mMTC devices are evenly distributed in a circular region where the radius is 200 m. All of the devices are served by the same base station that controls the allocation of subcarriers. This paper considers a flat Rayleigh fading channel. The distance-dependent path loss PL(D) is [12]

(17) PL(D)=120.9+37.6log(D/1000)+L+AG,

where D is the communication distance and AG is the antenna gain. The value of AG is 0.4 dB. *L* is the indoor penetration loss. we assume that 80% of mMTC equipment is indoor equipment, where *L* takes 20 dB; 20% is outdoor mMTC equipment, where *L* takes 0 dB.

**Figure 2:**
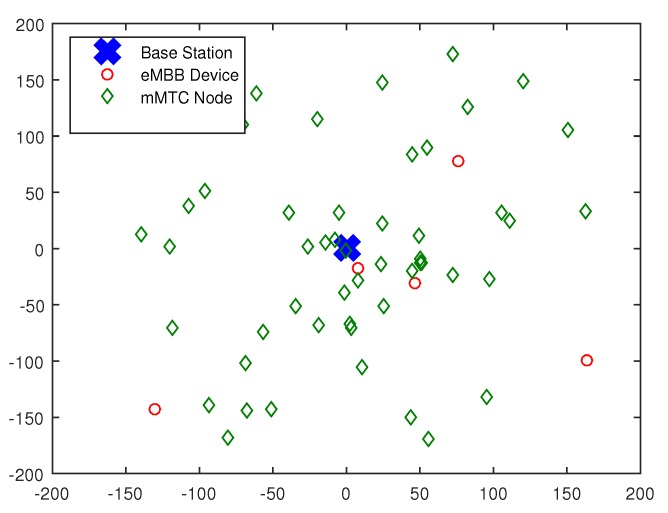
Distribution of eMBB devices and mMTC devices in cellular systems.

Figure 2 is a location distribution diagram of eMBB devices and mMTC devices. We consider an area with a radius of 200 m, in which the eMBB devices and mMTC devices are evenly and randomly distributed in the area, and the base station is set at the origin.

**Figure 3:**
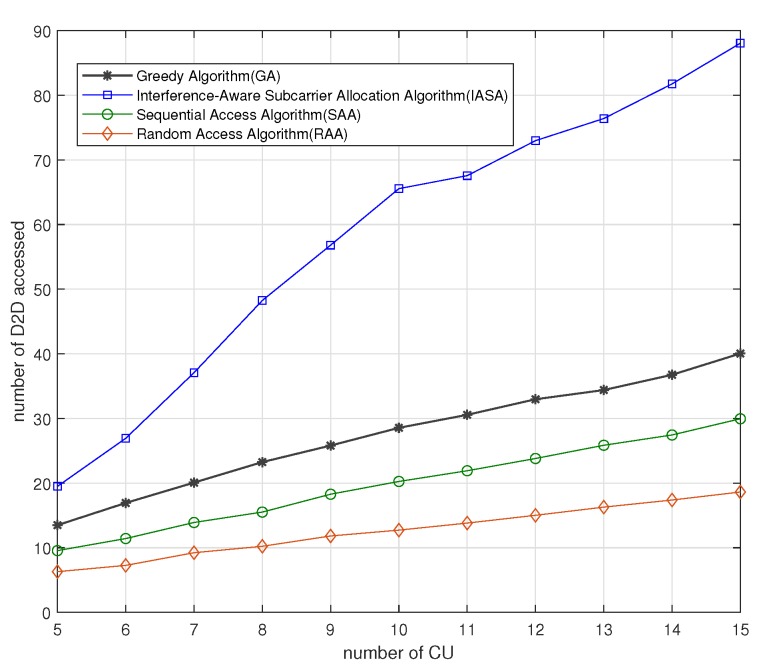
Number of mMTC pairs accessed versus different eMBB devices using different algorithms.

Figure 3 shows the number of mMTC pairs that successfully access subcarriers for different eMBB devices with $P_{i}^{c}=10$ dB, $P_{j}^{d}=7$ dB, $R_{cu_{-}\min}^{i}=10$ bps, $R_{j}=5$ bps. The total number of mMTC pairs is 150, and the interference range of the mMTC pair is 80 m. Among the four algorithms, the number of mMTC pairs accessed increases as the number of subcarriers increases. The reason is that when the number of subcarriers increases, the mMTC pairs will be more likely to access the subcarriers. Some mMTC pairs with large interference also have the opportunity to access the subcarriers. Compared to the three contrastive algorithms, the IASA algorithm can realize more mMTC devices accessed in the system. The reason is that under the premise of guaranteeing the QoS of the eMBB devices and the mMTC pairs, the mMTC pair with the least interference to the subcarriers is selected first according to the IASA algorithm. The distance limitation is established to reduce the interference between the adjacent mMTC pairs. Meanwhile, the complexity of the algorithm is reduced. For GA, each subcarrier gives priority to the access number optimization of itself rather than the access performance of the whole system, so the access number of the system cannot be maximized. However, since GA considers the mMTC priority access with less interference in each subcarrier, its performance is better than RAA and SAA. For RAA and SAA, there may be an mMTC pair with large interference accessing the subcarrier at any time, which occupies a large proportion of the interference space that the subcarrier can sustain. Under these circumstances, some mMTC pairs with small interference cannot access the subcarrier because the space for interference is finite. Therefore, the IASA algorithm enables the system to accommodate more mMTC pairs.

**Figure 4:**
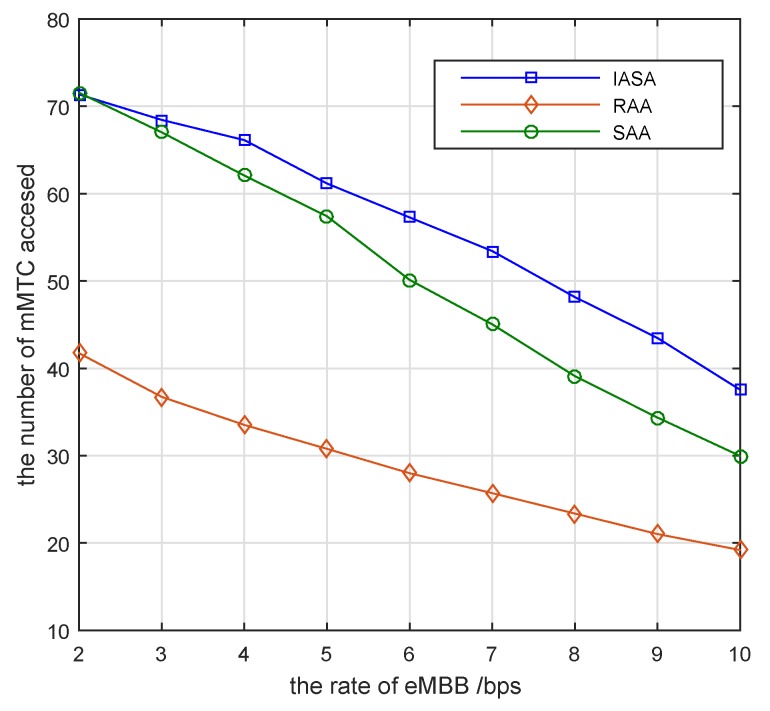
Number of mMTC pairs accessed versus different rate requirements of eMBB devices.

Figure 4 depicts the number of mMTC pairs that access subcarriers for different eMBB devices with $P_{i}^{c}=10$ dB, $P_{j}^{d}=7$ dB, $R_{j}=5$ bps. The interference range of the mMTC pairs is 80 m. The number of mMTC pairs that access the subcarriers decreases gradually when the minimum transmission rate of the eMBB devices gradually increases. The reason is that when the minimum transmission rate of the eMBB devices increases, the maximum interference that each subcarrier can sustain is reduced. In the case where the interference caused by the mMTC pair is unchanged, the number of mMTC pairs that can access the subcarriers is reduced. In the low-rate phase, the RAA exhibits much lower access performance than the IASA algorithm. And the SAA exhibits a comparable access performance with IASA algorithm. However, as the rate of eMBB devices increase, the performance of SAA have dropped significantly compared to the IASA algorithm.

**Figure 5:**
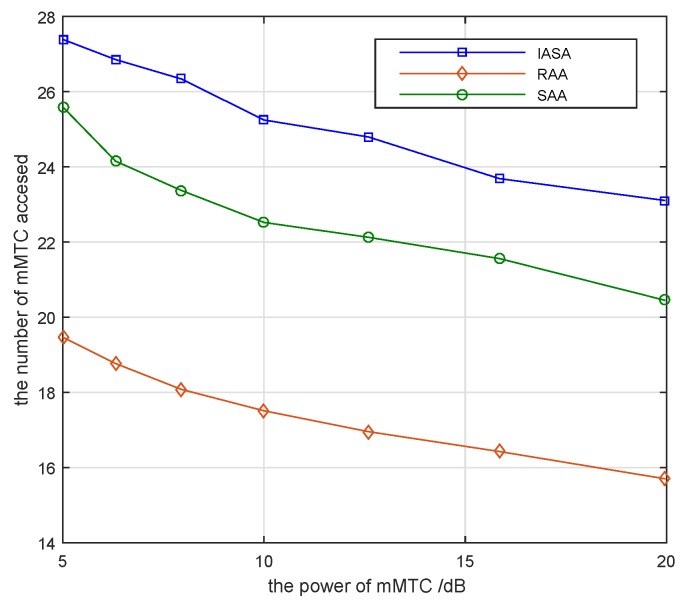
Number of mMTC pairs accessed versus different mMTC device transmit powers.

Figure 5 presents the number of mMTC pairs accessed when the power of the mMTC pairs transmitter change with $P_{i}^{c}=10$ dB, $R_{cu_{-}\min}=10$ bps, $R_{j}=5$ bps. The interference range of the mMTC pairs is 80 m. The number of accessed mMTC devices gradually decreases as the power of the mMTC transmitter increases. This is due to the fact that the interference to subcarriers increases while the power of the mMTC transmitting increases. Therefore, the number of mMTC devices accessed is relatively reduced. Compared to the variables such as the number of eMBB devices and the minimum transmission rate of eMBB devices, the power of the mMTC transmitter has a relatively small impact on the number of mMTC pairs accessed in the system.

**Figure 6:**
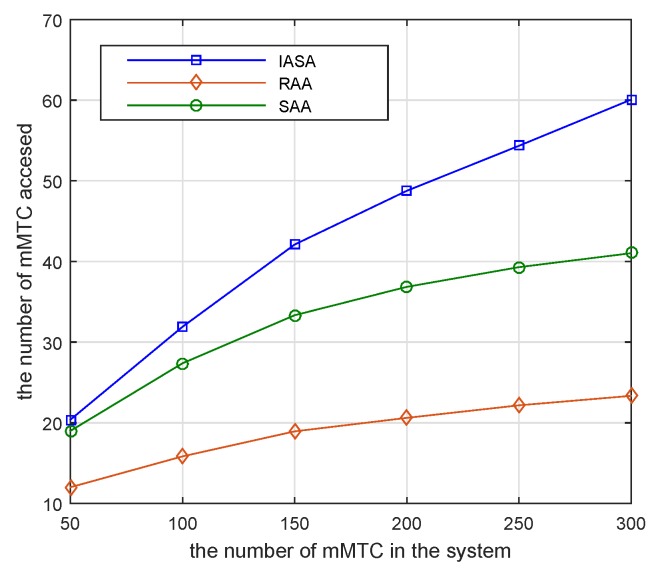
Number of mMTC pairs accessed versus different number of mMTC devices.

Figure 6 investigates the number of mMTC pairs accessed when there are different numbers of mMTC pairs in the system. The parameter settings are the same as in Figure 3. The number of mMTC devices accessed increases as the number of mMTC pairs in the system increases. Before the number of subcarriers is saturated, the more mMTC devices in the system, the greater the opportunity to access devices that satisfy the QoS requirements of the eMBB devices. Thus, the total number of mMTC pairs accessed will increase. However, when the power and the minimum transmission rate of the eMBB devices are fixed, the number of mMTC devices accessed by all subcarriers is constant, so the number of mMTC pairs accessed will gradually become saturated. Among the three algorithms shown in Figure 6, the RAA achieves the access saturation state first.

## 6. Conclusions

In this paper, an interference-aware subcarrier allocation algorithm for mMTC is proposed for the subcarrier allocation problem of D2D communication in mMTC scenarios. Initially, we establish a model maximizing the number of mMTC pairs accessed. When carrying out subcarrier allocation, we calculate the maximum interference that each subcarrier can sustain. Then we determine the mMTC pair with the least interference to the subcarrier and estimate the access property according to whether the QoS of the mMTC pair is satisfied. When the accumulated interference caused by the mMTC pair is greater than the maximum interference limit that the subcarrier can sustain, the subcarrier will be not be accessible to other mMTC pairs anymore. Simulation results demonstrated the effectiveness of the proposed algorithm.

## Figures and Tables

**Table 1:** System parameters.

Notation	Description
$g_{j}$	Gain between $MU_{j}^{t}$ and $MU_{j}^{r}$
$g_{i,B}$	Gain between $CU_{i}$ and the centralized controller
$h_{i,j}$	Interference gain between $CU_{i}$ and $MU_{j}^{r}$
$h_{j,B}$	Interference gain between $MU_{j}^{t}$ and the centralized controller
$f_{j^{\prime },j}$	Interference gain between $MU_{j^{\prime }}^{t}$ and $MU_{j}^{r}$
$P_{i}^{c}$	The transmit power of $CU_{i}$
$P_{j}^{d}$	The transmit power of $MU_{j}^{t}$
